# Energy, Sugars, Iron, and Vitamin B12 Content of Commercial Infant Food Pouches and Other Commercial Infant Foods on the New Zealand Market

**DOI:** 10.3390/nu13020657

**Published:** 2021-02-18

**Authors:** Ioanna Katiforis, Elizabeth A Fleming, Jillian J Haszard, Tiana Hape-Cramond, Rachael W Taylor, Anne-Louise M Heath

**Affiliations:** 1 Department of Human Nutrition, University of Otago, PO Box 56, Dunedin 9054, New Zealand; ioanna.katiforis@otago.ac.nz (I.K.); liz.fleming@otago.ac.nz (E.A.F.); jill.haszard@otago.ac.nz (J.J.H.); hapti994@student.otago.ac.nz (T.H.-C.); 2 Department of Medicine, University of Otago, PO Box 56, Dunedin 9054, New Zealand; rachael.taylor@otago.ac.nz

**Keywords:** infant food pouch, infant, nutrition, complementary feeding, commercial infant food, energy, iron, total sugars, free sugars, added sugars, vitamin B12

## Abstract

There has been an important shift in the New Zealand infant food market over the past decade, with the majority of complementary foods now sold in “pouches”. Along with the increasing market share of commercial infant food pouches internationally, there have been growing concerns about their nutritional quality. However, research examining the nutritional quality of these pouches compared to other forms of commercial infant foods in New Zealand has not been undertaken. Nor have any studies reported the free sugars or added sugars content of these foods. To address this knowledge gap, a cross-sectional survey of infant foods sold in New Zealand supermarkets was conducted in 2019–2020. Recipes and nutrient lines were developed for the 266 foods identified (133 food pouches). The energy, iron, vitamin B12, total sugars, free sugars, and added sugars content of infant food pouches and other forms of commercial infant foods per 100 g were compared, both within food groups and by age group. Infant food pouches contained similar median amounts of energy, iron, and vitamin B12 to other forms of commercial infant foods but contained considerably more total sugars (8.4 g/100 g vs. 2.3 g/100 g). However, median free sugars and added sugars content was very low across all food groups except for “dairy” and “sweet snacks”. All “dry cereals” were fortified with iron whereas none of the infant food pouches were. Therefore, consuming food pouches to the exclusion of other commercial infant foods may place infants at risk of iron deficiency if they do not receive sufficient iron from other sources.

## 1. Introduction

Good nutrition in infancy is vital for normal growth and development [1,2], and dietary habits established in infancy can have health implications throughout the life course [3]. For the first 6 months of age, the nutritional requirements of infants are met from breast milk or infant formula [2]. However, after 6 months of age, the infant diet must be supplemented by solid foods in order to meet the increased nutritional demands of later infancy [2]. These foods are known as “complementary foods” because they complement the infant’s milk-based diet. According to the New Zealand Ministry of Health (Wellington, New Zealand), the most important nutrients during the complementary feeding period are energy, iron, zinc and vitamin C [2].

The aim of complementary feeding is to transition the infant from an entirely milk diet to eating family foods by approximately 24 months of age [1]. Infant feeding guidelines developed by the World Health Organization (WHO) and the New Zealand Ministry of Health recommend the introduction of nutrient-rich complementary foods from 6, or “about” 6, months of age [1,2]. The guidelines aim to meet the nutrient requirements of infants during the complementary feeding period whilst also increasing their exposure to different tastes and textures to prepare them for a more mature diet [1,2].

Traditionally, complementary foods were prepared at home and were fed to infants with spoons [4]. Over the last decade, many countries around the world have seen a rapid growth in their commercial complementary food market, which has been attributed to urbanisation and higher female labour force participation [5]. As quoted by Padarath et al. [6], between 2010 and 2016, the commercial baby food sector in New Zealand grew by 27.2% and had a retail value of (NZD) $42.6 million in 2019 [6], while between 2013 and 2018, the retail value of the Australian baby food industry doubled from (AUD) $537.8 million to (AUD) $1.2 billion [7]. The products available on the New Zealand infant food market are much more diverse than the puréed fruits and vegetables sold in glass jars of past times. Complementary foods are also now sold in diverse forms, and the majority are sold in innovative, single-use squeeze food pouches with plastic nozzles (i.e., “pouches”). It has been reported that more than half of infant and toddler foods in the United States of America (USA) are packaged in pouches [8]. The potential benefits of complementary feeding with pouches include shorter meal times and convenience [9,10] particularly when feeding takes place outside of the home, because the foods are ready-to-eat and can be fed to infants without the need for eating utensils such as spoons. Although no research to date has investigated how food in these pouches is fed to infants, anecdotal reports suggest that infants commonly feed themselves by holding the pouch and squeezing or sucking the food through the nozzle.

Concerns have been raised by advocacy groups, community organisations, dietitians, and journalists about the nutritional quality of infant food pouches, and particularly their energy, sugars, and iron content [11,12,13,14,15,16]. In addition, a study investigating the nutrient intakes of infants following another recent baby-feeding approach (baby-led weaning) found that infants who were feeding themselves had lower intakes of vitamin B12 than traditionally spoon-fed infants [17]. Research examining the nutritional quality of pouches compared to other forms of commercial infant foods has not been undertaken in New Zealand, and little has been published about the nutritional quality of pouches internationally. Although previous studies have reported the total sugars content [8,18,19] and the percentage of energy from total sugars [8,18] in pouch foods, none have reported their free or added sugars content. Given the popularity and wide availability of pouches on the commercial infant food market, it is important to examine their nutritional quality.

Therefore, the aim of the study was to describe the energy, iron, vitamin B12, total sugars, free sugars, and added sugars content of foods in commercial infant food pouches compared to other forms of commercial infant foods sold by supermarkets in New Zealand.

## 2. Materials and Methods

### 2.1. Data Collection

Information was collected on infant foods commercially available in New Zealand in December 2019 to February 2020. Commercial infant foods were defined as foods sold in the “baby foods” section of the supermarket. Two authors (I.K. and T.H.-C.) visited the “baby foods” sections of four major supermarkets from the two major New Zealand supermarket chains (Foodstuffs (i.e., New World, PAK’nSAVE), and Woolworths (i.e., Countdown, FreshChoice)). Photographs were taken of the front of the packet, ingredient list, and nutrition information panel of each food. Infant formulas found in the “baby foods” sections were excluded because they are regulated by a separate standard of the Australia New Zealand Food Standards Code [20].

To ensure that all infant foods available in these New Zealand supermarkets were accounted for, an online search for “baby food” was conducted on the supermarket websites. If a food appeared on the website that was not on supermarket shelves at the time of the supermarket visit, the food’s ingredient list and nutrition information panel were obtained from the food manufacturer’s website. The following details for each food were entered into a Microsoft Excel (Microsoft Corporation, Washington, DC, USA) spreadsheet: form of food, brand, range, name, ingredients, and target age for consumption.

### 2.2. Nutrient Assessment

A recipe was developed for each food based on its ingredient list and the nutrient amounts per 100 g reported on its nutrition information panel. Dried herbs and spices were excluded from recipes because they do not contribute substantial weight or nutrients. The recipes were developed using FoodWorks 10 (FoodWorks 10 Professional, v10.0, Xyris Pty Ltd., Brisbane, Australia, 2019). FoodWorks is a nutrient analysis software package designed for use in Australia and New Zealand. The nutrient data in FoodWorks were from FOODfiles 2018 (the reference food composition table for New Zealand; jointly owned by the New Zealand Institute for Plant and Food Research Limited and the New Zealand Ministry of Health). “Free sugars” and “added sugars” are new additions to FOODfiles in the 2018 release [21]. The “free sugars” values were used for the current study but with fruit purées excluded so that the data were consistent with the WHO definition of “free sugars”, which is “monosaccharides and disaccharides added to foods by the manufacturer, cook or consumer, plus sugars naturally present in honey, syrups, fruit juice, and fruit juice concentrates” [22].

The USA does not use the term “free sugars” so the United States Food and Drug Administration (US FDA, Washington, DC, USA) definition of “added sugars” was also used. The US FDA definition of “added sugars” is sugars (free, monosaccharides and disaccharides), sugars from honey and syrups, and sugars from concentrated fruit and vegetable juices that are in excess of what would be expected from the same volume of 100% fruit or vegetable juice of the same type [23]. Therefore, the US FDA definition of “added sugars” differs from the WHO definition of “free sugars” in that the US FDA excludes sugars from concentrated fruit juice or vegetable juices that have been diluted to the same concentration as 100% fruit or vegetable juice of the same type, and some sugars found in fruit and vegetable juices, jellies, jams, preserves, and fruit spreads [23]. In accordance with the US FDA definition of “added sugars”, fruit or vegetable juice concentrates were therefore considered “added sugars” if they had not been reconstituted to 100% juice. We considered that fruit or vegetable juice concentrates present in foods where water was *not* a listed ingredient were “added sugars”. For reasons of practicality, where water was a listed ingredient, we considered that the fruit or vegetable juice concentrate had been reconstituted to 100% juice, because it was not possible to determine the strength that the concentrate had been reconstituted to from the information on the food label.

Ingredients were entered into FoodWorks as cooked ingredients. Where ingredients listed on the food labels were not available in FoodWorks, a nutritionally similar substitute ingredient was used, if there was one available (*n* = 33). If a nutritionally similar substitute was not available, and it was unlikely that the ingredient would substantially alter the nutrient values, it was excluded (*n* = 4). Infant cereals sold as dry cereals were entered in their prepared form (reconstituted with water), according to the manufacturers’ directions on the food label. To generate nutrient lines that were as accurate as possible, the aim when developing the recipe from the ingredient list was to ensure that the energy, fat, saturated fat (if reported), carbohydrate, and total sugars amounts were within 10% of the per 100 g amounts reported on the nutrition information panel. Nutrient amounts reported on nutrition information panels as less than an exact quantity (e.g., <1 g) were rounded to the nearest whole number for analysis. To account for fortification, amounts of iron that were automatically calculated in FoodWorks were overridden with the amount reported on the nutrition information panel.

Nutrient amounts were determined per 100 g so that differences in manufacturer-proposed serving sizes would not impact the results. The suggested portion sizes of the foods varied widely, even for similar foods with the same form; therefore, reporting nutrient amounts per 100 g ensured that differences in nutrient composition were due to actual differences among the foods rather than differences in manufacturer-proposed serving sizes.

### 2.3. Classification of Foods into Forms

Foods were classified into one of the following five forms, based on the form in which they were packaged and/or sold: “pouches”, “cans, dips, jars and pouch meals”, “dry cereals”, “snack foods”, and “tray/bowl meals”. Foods sold in pouches without nozzles were not classified as “pouches” because they could not be consumed directly from the pouch (i.e., they were assumed to be used in a similar way to non-pouch foods). Instead, these foods were classified as “pouch meals”.

### 2.4. Classification of Foods into Food Groups

Eight food groups were developed that covered all foods, while differentiating among iron sources (a nutrient of particular concern in this age group [24]), and allowing “like” foods to be compared with “like” (e.g., a “snack food” category was needed to ensure legume-based crisp snacks were not compared with hummus) (see Figure S1). Foods were classified into food groups using the following hierarchy: “snack food”, “meat and fish”, “legume”, “breakfast cereal”, “dairy”, “vegetable”, “fruit”, and “cereal, grains and pasta”. Snack foods were further classified into “sweet snack” or “savoury snack” groups according to whether the name suggested it had a sweet or savoury flavour. Fruit-only snack foods were considered “sweet snacks”, vegetable-only and all other snack foods were considered “savoury snacks”. Ingredient lists were consulted for snack foods with names that implied they contained a mixture of fruits and vegetables. For example, if the name of a rice-based crisp snack implied it contained both apple and leek, the ingredient list was reviewed to determine whether the snack food contained a higher proportion of apple or leek. If it contained a higher proportion of apple, it was classified as a “sweet snack”, but if it contained a higher proportion of leek, it was classified as a “savoury snack”.

For non-snack foods, foods that contained ingredients from a single food group were classified into the relevant food group. If a food contained a combination of ingredients from more than one food group, it was classified into a food group based on a hierarchical classification system (see Figure S1). First, the name of the food product was reviewed to determine whether it suggested that the food belonged to a specific food group (see Table 1). Then, the ingredient list was checked to determine whether the food product contained at least 5% of the named food. If the 5% requirement was met, the food product was classified into the food group. If the food product contained less than 5% of the food, it was classified into the food group to which the first non-water ingredient listed on the ingredient list belonged.

For some food products, the proportion of the food stated in the name was not listed on the ingredient list. The Australia New Zealand Food Standards Code requires percentage labeling information be provided only for ingredients and components that are considered by the manufacturer to be “characterising” of the food product [25]. In these cases, it was assumed that the food product contained at least 5% of the food as it was not possible to determine the quantity.

One author (I.K.) classified the foods into food groups. A second author (E.A.F.), a registered dietitian, independently classified the foods into food groups. Then, the two authors discussed their results and resolved discrepancies in their classifications.

### 2.5. Classification of Foods into Target Age Groups

Foods were classified into four age groups (4–6 months, 6–8 months, 8–12 months, 12+ months) based on the minimum recommended age specified on the food label (see Table 2). A wide range of age groups were stated by manufacturers, so the four age groups used in the analysis were based on the developmental stages in the Food and Nutrition Guidelines for Healthy Infants and Toddlers developed by the New Zealand Ministry of Health [2]: foods recommended for infants from 4 or 5 months of age were classified into the 4–6 month age group, foods recommended for infants from 6 or 7 months of age were classified into the 6–8 month age group (encompassing the 6–7 month and 7–8 month developmental stages [2]), foods recommended for infants from 8, 9, 10, or 11 months of age were classified into the 8–12 month age group, and foods recommended for infants 12 months of age or over were classified into the 12+ month age group. If the label did not specify a recommended age (*n* = 2), the food was classified into the same age group as other similar foods.

The label of one food (soft cereal biscuits) specified two recommended ages, which differed depending on whether or not the biscuit was eaten as is or dissolved. It was classified into the 8–12 month age group, as this was the most convenient way to consume the food (i.e., as a biscuit).

### 2.6. Statistical Analysis

All descriptive statistics, tables, and figures were generated using Stata v16 (StataCorp, College Station, TX, USA). Box plots were created in Stata to show the distribution of nutrient values. It was decided to use box plots to present the data visually rather than presenting results from statistical tests because box plots display all of the data, allowing for transparency when making conclusions about the significance of the results, rather than relying on *p*-values for interpretation, which have limitations and can be misleading [26]. Median values were calculated in Stata and displayed above the boxes.

The line inside the boxes indicates the median (mid-point) of the data. The boxes indicate the interquartile range (IQR), which is the distribution of the middle 50% of the nutrient values, ranging from the lower 25th percentile to the upper 75th percentile. The lower whiskers contain the lower 0–25% of the nutrient values and the upper whiskers contain the higher 75–100% of the nutrient values. Outliers are indicated by the circles, which signify data points that are more than 1.5 times the IQR.

Results were also stratified in Figure 2 by the separate forms in which foods were packaged and/or sold (“pouches”, “cans, dips, jars and pouch meals”, “dry cereals,” and “tray/bowl meals”) so that differences in the nutrient content among the different forms of “non-pouch” foods could be seen. Furthermore, results were stratified by food group and age group so that differences in the nutrient content of “pouch” versus “non-pouch” foods could be seen within food groups and by age group. The nutrient content of snack foods (stratified into “sweet snacks” and “savoury snacks”) was analysed separately because they were all designed to be consumed in a dry form, so they had a substantially higher nutrient concentration than the rest of the foods.

## 3. Results

### 3.1. General Characteristics of Commercial Infant Foods

A total of 266 foods were found across 19 brands. Of these, 58 foods were classified as “snack foods” and were excluded from the main nutrient analyses but are reported separately.

Of the 208 non-snack foods found (see Table 3), 133 (63.9%) were sold in pouches and 75 (36.1%) were sold in other forms of packaging (i.e., “non-pouches”). Of the “non-pouches”, 58 (77.3%) foods were classified as “cans, dips, jars and pouch meals”.

Fifty-three (25.5%) foods overall were classified as “fruit” (48 of these were pouches). Fifty-one (24.5%) foods were classified as “meat and fish”, but only 18 of these were pouches (13.5% of all pouch foods). Overall, the majority of pouches were “fruit” (36.1%), followed by “vegetable” (21.1%) and “dairy” (18.0%).

Of the 208 foods, 43 (20.7%) foods were targeted at infants 4–6 months of age (35 of these were pouches), with 88 (42.3%) targeted at infants 6–8 months of age (72 of these were pouches). Twenty (9.6%) foods were targeted at infants 12+ months of age (7 of these were pouches). Overall, the majority of the 133 pouches were targeted at infants 6–8 months of age (54.1%), followed by infants 4–6 months of age (26.3%).

Of the 58 snack foods, 38 (65.5%) were classified as “sweet snacks” and 20 (34.5%) were classified as “savoury snacks”.

### 3.2. Energy, Iron, Vitamin B12, and Sugars Content of Pouches Vs. Non-Pouches 

**Figure 1:**
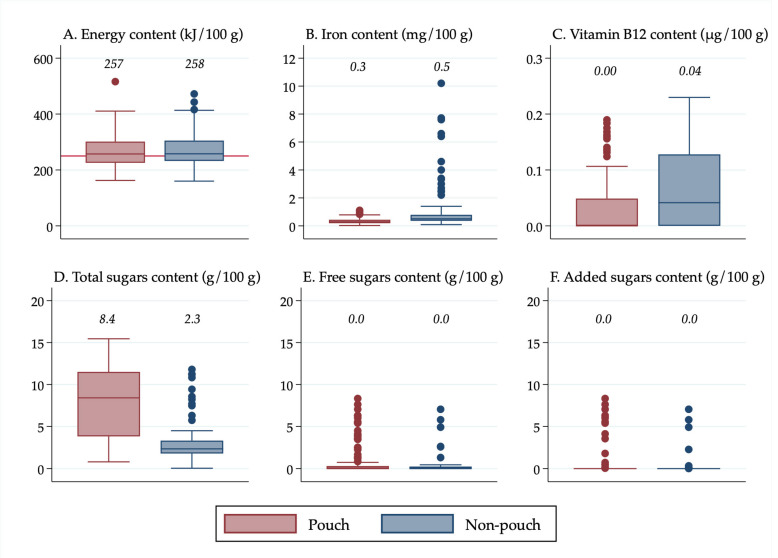
(**A**) Energy content of foods in pouches vs. non-pouches; (**B**) Iron content of foods in pouches vs. non-pouches; (**C**) Vitamin B12 content of foods in pouches vs. non-pouches; (**D**) Total sugars content of foods in pouches vs. non-pouches; (**E**) Free sugars content of foods in pouches vs. non-pouches; (**F**) Added sugars content of foods in pouches vs. non-pouches. Median values are displayed above the boxes. The line at the 250 kJ/100 g point in Figure 1A indicates the minimum energy density proposed by the WHO in their criteria for commercially available complementary foods that are considered suitable for infants and young children [27]. See Figure S2 for energy content in kcal.

The median energy, iron, and vitamin B12 content of “pouches” were similar to “non-pouches” when the non-pouch foods were combined, but “pouches” contained more than three times as much total sugars as “non-pouches” (median 8.4 g/100 g vs. 2.3 g/100 g) (Figure 1). The “non-pouch” outliers for iron content (Figure 1B) were prepared “dry cereals”. “Pouch” outliers for vitamin B12 (Figure 1C) were predominantly “meat and fish” pouches. Neither “pouches” nor “non-pouches” contained free or added sugars on average. All of the free and added sugars outliers (Figure 1E,F) that contained greater than 5 g/100 g of free or added sugars were custards.

### 3.3. Energy, Iron, Vitamin B12 and Sugars Content by Form of Food

**Figure 2:**
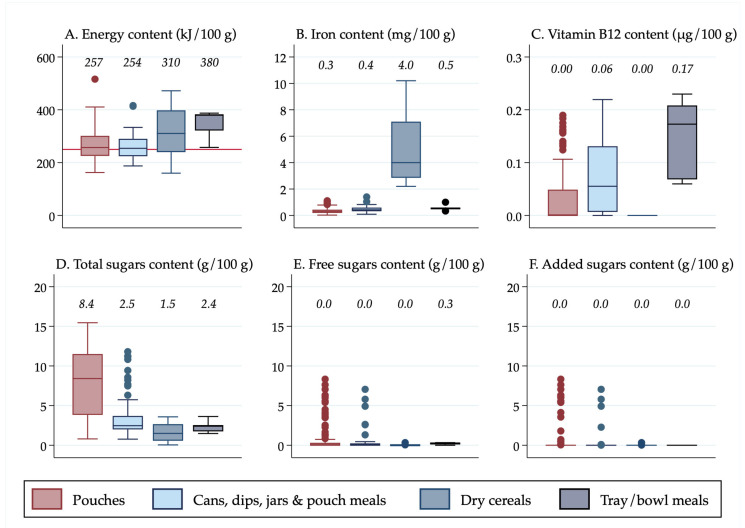
(**A**) Energy content of foods by form of food; (**B**) Iron content of foods by form of food; (**C**) Vitamin B12 content of foods by form of food; (**D**) Total sugars content of foods by form of food; (**E**) Free sugars content of foods by form of food; (**F**) Added sugars content of foods by form of food. ^1^ Median values are displayed above the boxes. ^2^ The line at the 250 kJ/100 g point in Figure 2A indicates the minimum energy density proposed by the WHO in their criteria for commercially available complementary foods that are considered suitable for infants and young children [27]. ^3^ See Figure S3 for energy content in kcal.

“Tray/bowl meals” had the highest median energy content (380 kJ/100 g), followed by prepared “dry cereals” when reconstituted (310 kJ/100 g) (Figure 2). All forms of foods, apart from prepared “dry cereals”, contained low amounts of iron. Prepared “dry cereals” contained more than 10 times as much iron as “pouches” (median 4 mg/100 g vs. 0.3 mg/100 g). “Tray/bowl meals” contained a median of 0.17 µg/100 g of vitamin B12 which was considerably higher than the other forms of foods.

The median total sugars content of “pouches” was more than three times that of “cans, dips, jars, and pouch meals” and “tray/bowl meals” (8.4 g/100 g vs. 2.5 g/100 g and 2.4 g/100 g) and more than five times that of prepared “dry cereals” (8.4 g/100 g vs. 1.5 g/100 g). On average, free sugars and added sugars were absent from all food forms, apart from “tray/bowl meals”, which contained a small amount of free sugars (median 0.3 g/100 g), although there were a number of outliers (14 of the 133 “pouches” had more than 1 g/100 g of free and added sugars, compared to four of the 58 “cans, dips, jars and pouch meals”).

### 3.4. Energy, Iron, Vitamin B12, and Sugars Content by Food Group

**Figure 3:**
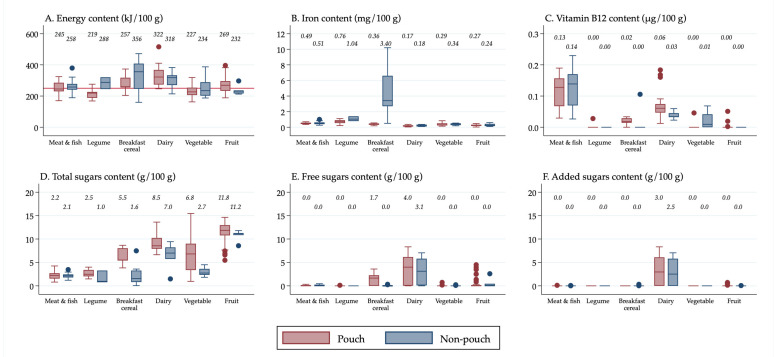
(**A**) Energy content of foods by food group; (**B**) Iron content of foods by food group; (**C**) Vitamin B12 content of foods by food group; (**D**) Total sugars content of foods by food group; (**E**) Free sugars content of foods by food group; (**F**) Added sugars content of foods by food group. ^1^ Median values are displayed above the boxes. ^2^ The line at the 250 kJ/100 g point in Figure 3A indicates the minimum energy density proposed by the WHO in their criteria for commercially available complementary foods that are considered suitable for infants and young children [27]. ^3^ The “Cereal, grains and pasta” food group is not shown, as only two foods were contained in the food group. This is because all other foods were classified into other food groups at higher levels of the classification system (see Figure S1). ^4^ See Figure S4 for energy content in kcal.

For each of the food groups, the median energy, iron, and vitamin B12 content of pouch and non-pouch foods was similar, except that non-pouch foods in the “breakfast cereal” food group contained considerably more iron than pouch foods (3.40 mg/100 g vs. 0.36 mg/100 g) (Figure 3). Overall, pouch and non-pouch foods contained low amounts of vitamin B12 across all food groups, although pouches and non-pouches in the “meat and fish” food group contained higher amounts (0.13 µg/100 g and 0.14 µg/100 g).

Across all food groups except “meat and fish”, pouch foods contained more total sugars than non-pouch foods. Pouches in the “fruit”, “dairy”, and “vegetable” food groups contained the most total sugars (median 11.8 g/100 g, 8.5 g/100 g, and 6.8 g/100 g respectively). “Breakfast cereal” pouch foods had slightly higher median free sugars content than non-pouch foods (median 1.7 g/100 g vs. 0.0 g/100 g). The “dairy” food group contained the most free sugars and added sugars, which was similar for pouch and non-pouch foods. Data on percentage of energy from free sugars are presented in Table S1. Of all the food groups, the “dairy” food group pouch and non-pouch foods contained the highest percentage of energy from free sugars (median 25.6% and 17.8%). On average, “breakfast cereal” pouch foods contained 7.4% of energy from free sugars, compared to 0.0% for “breakfast cereal” non-pouch foods.

### 3.5. Energy, Iron, Vitamin B12 and Sugars Content by Target Age Group 

**Figure 4:**
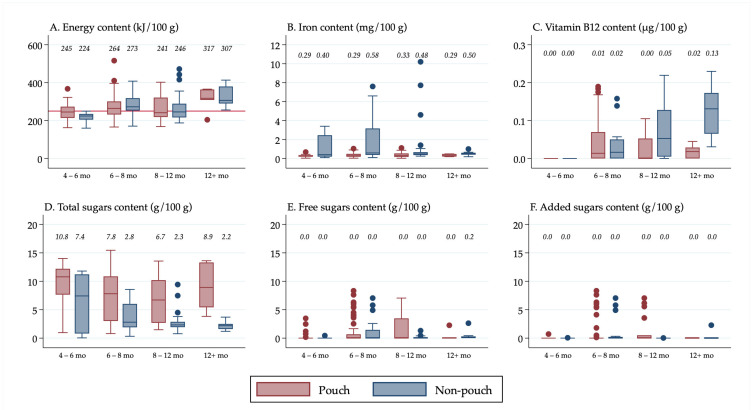
(**A**) Energy content of foods by age group; (**B**) Iron content of foods by age group; (**C**) Vitamin B12 content of foods by age group; (**D**) Total sugars content of foods by age group; (**E**) Free sugars content of foods by age group; (**F**) Added sugars content of foods by age group. ^1^ Median values are displayed above the boxes. ^2^ The line at the 250 kJ/100 g point in Figure 4A indicates the minimum energy density proposed by the WHO in their criteria for commercially available complementary foods that are considered suitable for infants and young children [27]. ^3^ See Figure S5 for energy content in kcal.

Overall, for each of the age groups, the median energy, iron, and vitamin B12 content of pouches compared to non-pouches did not differ widely, except that vitamin B12 content was almost seven times higher for non-pouch than pouch foods in the 12+ month age group (0.13 µg/100 g vs. 0.02 µg/100 g) (Figure 4). The “non-pouch” outliers for iron content (Figure 4B) in the 8–12 month age group were prepared “dry cereals”. Neither pouch nor non-pouch foods in the 4–6 month age group contained any vitamin B12.

Pouches contained more total sugars than non-pouches across all age groups. Although on average neither pouches nor non-pouches contained free sugars and added sugars, there were a number of outliers, particularly at 6–8 months (14 “pouch” outliers for free sugars content (Figure 4E) and 14 “pouch” outliers for added sugars content (Figure 4F).

### 3.6. Energy, Iron, Vitamin B12, and Sugars Content by Snack Food Type 

**Figure 5:**
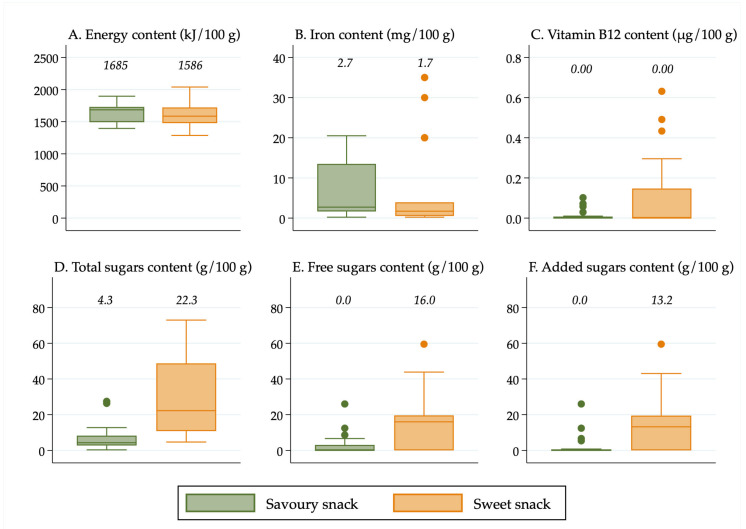
(**A**) Energy content of snack foods; (**B**) Iron content of snack foods; (**C**) Vitamin B12 content of snack foods; (**D**) Total sugars content of snack foods; (**E**) Free sugars content of snack foods; (**F**) Added sugars content of snack foods. ^1^ Median values are displayed above the boxes. ^2^ See Figure S6 for energy content in kcal.

“Savoury snacks” and “sweet snacks” contained similar median amounts of energy, iron, and vitamin B12 (Figure 5). The “sweet snack” outliers for iron content (Figure 5B) were iron-fortified snack foods. Neither “savoury snacks” nor “sweet snacks” contained any vitamin B12 on average, although there were 12 “sweet snacks” that contained greater than 0.1 µg/100 g. The median total sugars content of “sweet snacks” was over five times that of “savoury snacks” (22.3 g/100 g vs. 4.3 g/100 g). One sweet snack product, a solid dairy “yoghurt” snack, contained 66.9 g/100 g of total sugars and 59.5 g/100 g of both free and added sugars. On average, 16.0% of the energy content of “sweet snacks” was from free sugars, whereas free sugars did not provide any of the energy content of “savoury snacks” (median 0.0%).

## 4. Discussion

Infant food pouches contained similar median amounts of energy, iron, and vitamin B12 to other forms of commercial infant foods but contained considerably more total sugars (8.4 g/100 g vs. 2.3 g/100 g). Interestingly, this was not the case for free and added sugars. Although there were some outliers, when the food groups were combined, on average, pouches did not contain any free or added sugars, and 79.7% of pouches contained less than 1.0 g/100 g of free sugars and 90.2% contained less than 1.8 g/100 g of added sugars. When the food groups were compared, the “dairy” food group (whether presented in a pouch or not) contained considerably more free sugars and added sugars than the other food groups. The iron content of all forms of commercial infant foods was very low, except for prepared “dry cereals”. This is because all of the prepared “dry cereals” were fortified with substantial amounts of iron. In contrast, none of the food pouches were fortified with iron. Of the 58 snack foods identified, 33 contained at least one source of free sugars and 30 contained at least one source of added sugars.

The current study found that food in pouches had a similar energy content to other forms of commercial infant foods. This suggests that concerns about the risk of excess energy consumption [12,13,28], if supported by future research, would not be from the energy content of pouch foods, but rather would be from the influence of pouches on eating behaviour. For example, this could be due to the ease with which pouch foods can be delivered and consumed, as the texture of the food in pouches must be smooth enough to fit through the nozzle. In addition, the soft walls of the pouch allow the food to pass rapidly through the nozzle when pressure is applied to the outside of the pouch [9], which may mean that parents or caregivers pour more of the food from the pouch than they intend, or that the infant eats a larger portion of pouch food, particularly if the speed of consumption outstrips mechanisms for identifying and signalling satiety. In New Zealand, food labels recommend that the pouch contents are poured onto a spoon or bowl to be fed to infants; however, anecdotal reports suggest that these recommendations are not being followed and infants are self-feeding from pouches. Certainly consumer research focus groups carried out in the United Kingdom (UK) and reported by Public Health England have revealed that some mothers allow their infants to self-feed directly from pouches by sucking the contents out through the nozzle, and that this was more likely to occur when mothers and infants were out of the home [29]. Research examining pouch use and possible associations with energy intake and body weight is currently being undertaken in New Zealand (Australia New Zealand Clinical Trials Registry (ANZCTR) registration number: ACTRN12620000459921).

The finding in the current study that pouch foods and non-pouch foods had similar energy content is also surprising, because it has been suggested that additional water is added to pouches to achieve the smooth consistency and loose texture required for the food to be squeezed out of the nozzle [30]. Accordingly, it may have been expected that pouch foods would contain less energy than non-pouch foods. However, for commercial infant foods in the New Zealand market, there was no evidence that more water was being added to pouches than to non-pouch commercial infant foods. In fact, a smaller proportion of pouch foods contained water as an added ingredient than non-pouch foods (excluding prepared “dry cereals” and snack foods) (65.4% compared to 79.4%), and similar proportions of pouch foods and non-pouch foods listed water as the first ingredient (i.e., the largest quantity ingredient) on the ingredient list (18.8% compared to 20.6%). Interestingly, the added water content of pouches, and of commercial infant foods generally, has been raised as an issue in the UK and Europe, because the addition of water lowers the energy density of a food, which may require that infants consume a greater volume of the food to meet their daily energy requirements [30,31]. Ultimately, the overall impact of consumption behaviour, and water and energy content, can only be determined by investigating the growth of infants who are using pouches and comparing it with the growth of those who are not.

The finding that pouches, on average, contained just 0.3 mg/100 g of iron is concerning because iron deficiency in infancy can affect growth and nervous system development [32], and by 6 months of age, infants’ iron stores are depleted. As a result, the need for iron increases substantially from 0.2 mg/day at 0–6 months of age (Adequate Intake (AI)) to 7 mg/day at 7–12 months of age (Estimated Average Requirement) (EAR)) [33]. In the current study, prepared “dry cereals” had the highest median iron content of all forms of commercial infant foods because they were all fortified with iron, with some dry cereals containing up to 10 mg/100 g of iron, whereas most pouches contained less than 1 mg/100 g of iron. Under the Australia New Zealand Food Standards Code, “cereal-based foods” (i.e., foods containing 70% or more cereal) that are promoted as being suitable for infants are required to be fortified with iron [34]. Presumably infant food pouches are not fortified with iron because their cereal content is diluted to less than 70% as they are pre-prepared, and because water-soluble iron compounds can cause undesirable changes in taste, colour, and texture, particularly in foods that are stored for extended periods of time [35], such as pouch foods. However, it is important that parents and caregivers do not assume that infant cereal in a pouch is nutritionally equivalent to a prepared dry infant cereal.

Despite prepared “dry cereals” containing only non-haem iron, which is less bioavailable (non-haem iron bioavailability in a diet that contains some meat is approximately 17%, compared to a haem iron bioavailability of approximately 25% [36]), prepared “dry cereals” were a much richer source of iron than pouches. The proportion of meat, fish, or poultry in the “meat and fish” pouches was only 5–10% (in non-pouches, it was 5–12%). “Meat and fish” pouches may be perceived by parents or caregivers as a rich source of iron, but they contain surprisingly little meat, fish, or poultry. It is possible that parents or caregivers feeding infants meat, fish, or poultry-containing homemade complementary foods offer larger amounts of meat, fish, or poultry than the amounts contained in pouches, if they offer family foods in puréed form. Unlike in the European Union, there are no regulations in New Zealand requiring that infant foods contain a minimum percentage of meat, fish, or poultry even when they are included in the name of the food.

The findings of the current study differ from those of Beauregard et al. [19], who reported that “meat-, fish-, or legume-based” and “yoghurt- or milk-based” pouches in the USA contained more iron than other packaging types. However, the differences found by Beauregard et al. [19] were small (ranging from 0.1 mg/100 g to 0.3 mg/100 g). The meat, fish, or poultry content of the foods examined in that study was not reported so it is not known how much meat, fish, or poultry contributed to the iron content. On the other hand, the findings of the current study are similar to those reported in an Australian survey of commercial infant and toddler foods, which found that most infant foods were poor sources of iron except for iron-fortified cereals [7]. Pouches were not examined separately in that survey.

Surprisingly, in the current study, 27.6% (*n* = 16) of snack foods were fortified with iron *(n* = 6 “savoury snacks”, *n* = 10 “sweet snacks”). The median snack serving size was 9 g; therefore, the iron content in one serving of fortified snacks with the highest amounts of iron (i.e., 35 mg/100 g), would equate to approximately 3 mg. However, it would be inappropriate to recommend snack foods as an iron source both because of the high sugars content of sweet snacks and because it is important not to encourage the development of eating patterns in which commercial snack foods are a regular part of the diet.

On average, pouches contained similar, very low, amounts of vitamin B12 to other forms of commercial infant foods. The AI for vitamin B12 for infants aged 0–6 months is 0.4 µg/day [33]; however, pouches targeted at infants in the 4–6 month age group contained no vitamin B12, and even pouches targeted at older infants in the 12+ month age group provided only 0.02 µg/100 g of vitamin B12 on average. “Tray/bowl meals” contained higher amounts of vitamin B12 as they had a higher proportion of meat, fish, or poultry, but these were only marketed to infants in the 12+ month age group. Infants require a reliable source of vitamin B12, and this would usually be obtained from breast milk or infant formula [2]. However, with the increasing interest in vegan diets, it is important that breastfeeding vegan mothers know that they need to consume adequate amounts of vitamin B12 from supplements or vitamin B12-fortified foods [2], and that commercial infant foods cannot be relied upon to supply this.

In the current study, pouches contained higher amounts of total sugars across all age groups and food groups than non-pouches, with pouches containing 6.1 g/100 g more total sugars on average. Sweet fruits and vegetables in puréed form were the primary contributors to the total sugars content of pouches, often being listed as the first ingredient. Of the 48 “fruit” pouches, the most frequently listed first ingredients were apple (*n* = 24) and pear (*n* = 14). Of the 28 “vegetable” pouches, over half (*n* = 16) listed a fruit as the first ingredient. Of the 12 vegetable-only pouches, over half (*n* = 8) listed kumara (a type of sweet potato), sweet potato, or pumpkin as the first ingredient. Koletzko et al. [18] reported that pouches available in Germany contained predominantly sweet, puréed fruit preparations, which were typically the source of the majority (or even all) of their sugars content. Moding et al. [8] reported that the high sugars content of pouches available in the USA is driven by the presence of fruits and vegetables that are naturally higher in sugars, as has been reported for commercial infant foods in general in the UK [37]. Moreover, a recent study of commercially available complementary foods in New Zealand reported that “relatively sweet” vegetables were used more frequently than “bitter” vegetables [6].

It has been suggested that food manufacturers use sweet vegetables in commercial infant foods because of commercial pressure to produce instantly palatable foods [37]. Infants have an innate preference for sweet tastes and dislike bitter tastes [38]. Adding sweet ingredients to pouches may improve palatability and mask more savoury vegetable tastes (e.g., broccoli or spinach). This is a concern because repeated exposure to vegetable flavours is needed to improve vegetable acceptability [39], and early and repeated exposure to sweet complementary foods may lead to infants being accustomed to consuming sweetened infant foods frequently, which could have health implications [40,41]. Foterek et al. [42] reported that a higher consumption of commercial complementary foods in infancy was associated with higher intakes of added sugar in later childhood. Concerns have been raised about parents and caregivers unknowingly exposing infants to sweet tastes more frequently than intended because of misleading labelling [37], with studies examining commercial infant food markets in the UK [29] and in several European countries [31] reporting misleading labelling. In New Zealand, the issue does not appear to be about disguising fruit in savoury meals but about the product name implying that the meal contains higher quantities of more nutritious foods than it in fact does. In the current study, a number of the food product names did not accurately reflect the proportions of ingredients contained in the food. For example, in a puréed product containing less than 5% spinach, “spinach” appeared first in the product name, implying that the largest quantity ingredient was “spinach” when it was in fact “apple” (which appeared second in the product name).

This is the first study to report the “free sugars” and “added sugars” content of commercial infant foods. The finding that pouches, on average, did not contain any free or added sugars was surprising, particularly given the widespread concern about high sugars content in pouches, and the emphasis being placed on the sugars content of commercial infant foods in the literature [8,28,43]. The WHO Regional Office for Europe made a call in 2019 to ban added sugars and “sweetening agents” (including fruit juices and fruit juice concentrates) in commercially available complementary foods [27]. A number of their proposed criteria for commercially available complementary foods that are considered suitable for infants and young children [27] are of particular relevance here. Meanwhile, the majority of pouch and other foods met the minimum energy density criterion of 250 kJ/100 g; there were 18 sweetened cows’ milk products (of which 14 were pouches) and 38 sweet snacks on the market where it is recommended that there should be none; total sugar content was greater than the maximum recommended 15% energy for two (10%) dry savoury foods; added sugars and other sweetening agents were present in 75 (28%) of foods (including 33 pouches) whereas it is recommended that sugars are not added to any infant foods; and puréed fruit was present in 24 (23%) savoury foods (21 pouches)—although some of these would have less puréed fruit than the amount that is of concern, i.e., <5% of total weight.

There is no universally agreed definition for sugars that “may have physiological consequences different from intrinsic sugars incorporated within intact plant cell walls or lactose naturally present in milk” [44]. While the WHO’s “free sugars” definition includes the sugars that are *added* to foods and beverages, it also includes the sugars naturally present in honey, syrups, fruit juices, and fruit juice concentrates [22]. In contrast, the US FDA’s “added sugars” definition excludes sugars from concentrated fruit and vegetable juices that have been diluted to single strength [23]. On this basis, sugars from a fruit juice ingredient in a pouch, for example, would not be included in the US FDA’s “added sugars” definition if it was diluted to single strength, but it would be included under the WHO’s “free sugars” definition because the WHO definition includes “fruit juices”. In addition to the WHO and US FDA definitions, a number of other definitions are used by researchers and commentators, with some using total sugars as a proxy. This is presumably because food composition databases have only recently provided “free sugars” and “added sugars” data, and because nutrition information panels for food products sold in New Zealand are required to report total sugars content only. The different definitions of sugars used in nutrition research studies and in dietary guidelines make comparisons between sugar content, sugar intake, and sugar recommendations difficult.

Importantly, current definitions of “free sugars” and “added sugars” are not tailored to account for foods typically consumed in the infant diet and typical preparation methods. For example, ingredients used in infant foods such as fruit or vegetable pastes, and fruit or vegetable powders, are not accounted for under the WHO and US FDA definitions of sugars, even though they provide sugars. Interestingly, the Scientific Advisory Committee on Nutrition (SACN) in the UK have proposed an extension to the WHO’s “free sugars” definition to include fruit and vegetable pastes, fruit and vegetable powders, as well as “fruit purées” and “vegetable purées” [45]. If the current study had used the SACN definition, then the “free sugars” content of pouches would have been much closer to the total sugars content, given the substantial proportion of pouches containing puréed fruits and vegetables. However, the SACN’s inclusion of purées in the definition of “free sugars” would have important implications for complementary feeding. At the current time, the WHO and the New Zealand Ministry of Health recommend the introduction of puréed foods from 6 months of age [1,2]. Fruits and vegetables are important parts of the diet at any age, and the New Zealand Ministry of Health lists puréed fruits and vegetables as an example of appropriate first complementary foods [2]. In fact, mashed foods and chopped foods are not recommended until later stages of the complementary feeding period because they may pose a choking risk in younger infants [2]. Although the First Steps Nutrition Trust in the UK propose that only sugars in *processed* fruit and vegetable purées are classified as “free sugars”, suggesting that this is because foods prepared at home are likely to have more of the sugars maintained within the cell wall [30], research is required to determine how home and commercial processing of fruit and vegetable purées impact their nutrient content and flavour profile, and the consequences this may have for infant health.

Even using the more widely agreed WHO and US FDA definitions, the current study identified a number of foods that are of particular concern. “Sugar”, as listed in the ingredient lists, was added to 13 dairy “pouches” and three dairy “cans, dips, jars and pouch meals”—all of them custards. The New Zealand Ministry of Health specifically recommends that infants do not consume complementary foods with added sugars, honey, and other sweeteners [2]. It is disappointing that food manufacturers are adding sugars to some infant foods, despite the Ministry of Health’s clear recommendations. In New Zealand, there is no maximum limit to the amount of added sugars that commercial infant foods may contain, although foods that contain greater than 4 g/100 g of added sugars must state that they are “sweetened” on the label [34]—this is inadequate and more stringent regulation of the composition and labelling of infant foods is required to ensure that parents and caregivers can clearly see when, and how much, “sugars” have been added to food products.

The WHO recommendations to limit free sugars intake to less than 10% (or 5% for additional health benefits) of total energy intake were made with an emphasis on preventing and controlling unhealthy weight gain and dental caries [22]. These are important issues even in infancy and early childhood. Globally, 38 million children under the age of five were overweight or obese in 2019 [46]. Moreover, excessive weight gain in childhood may lead to an increased risk of obesity, type 2 diabetes, cardiovascular disease, and some types of cancer in adulthood [47]. Dental caries is the most widespread non-communicable disease globally [22], and the condition may develop as early as six months of age, when the primary teeth erupt [48]. Dental caries in primary teeth can lead to pain and poor appetite which impact on sleep, well-being, and may affect an infant’s growth [49].

However, it is important to note a number of caveats. First, some commentators have expressed concerns about the “high sugars content” of pouches (relying on the total sugars amount reported on the label) and pointed to the use of fruit as a particular concern [11,28]. Although fruits do contain sugars, unlike table sugars, they also provide beneficial nutrients including phytochemicals, antioxidants, and dietary fibre [50]. For example, fruit purées contain dietary fibre, which is absent in table sugar. The amounts of nutrients may not be as high as for the whole fruit (particularly if the purée uses peeled fruit, which would reduce fibre content [51]; is cooked, which would reduce the content of heat-labile nutrients such as vitamin C [52]; or is drained before packaging, which would reduce soluble vitamin and mineral content), but the food may still be useful, particularly at this age when many whole fruits are not safe for infants to eat because of the choking hazard. Second, the way in which foods that contain sugars are consumed is also important, particularly for dental health—A sweet food that is consumed as part of meal that includes other foods or drinks that are not high in carbohydrate will have much less impact than a sweet food that is consumed repeatedly or over a prolonged period of time (for example, a food that is sipped). Third, many homemade foods are likely to also have sugars added to them—few parents or caregivers would prepare custards or similar dairy foods without adding some sugars. Ultimately, the amount of sugars, even total sugars, in commercial infant foods available in New Zealand is not high (particularly in the context of a diet that is predominantly breast milk or infant formula), with the exception of sweet snacks, dairy foods, and perhaps fruits.

The data in the current study are reported per 100 g, but it is important to remember that the amount of energy, sugars, and other nutrients consumed by a child is impacted by the amounts of food they are offered. Existing data suggest that the self-regulation of energy intake may not be as effective once a range of solid foods are being consumed as it is while the diet comprises infant milk only [53]. Yet, manufacturer-suggested portion sizes differ markedly for all product types. For example, suggested portion sizes for “pouches” ranged from 60 to 150 g, and those for “cans, dips, jars and pouch meals” ranged from 6 to 220 g. Some pouch and other packages contained two portions per package. It is unlikely that parents weigh out exact manufacturer-proposed serving sizes from these multi-serving packages to offer their child, and presumably, there are some parents who assume that a package of infant food provides an appropriate amount of food for an infant of a particular age. Ultimately, the Australia New Zealand Food Standards Code does not define serving sizes or reference quantities for food manufacturers to adhere to when suggesting to parents how much would be an appropriate amount of a product to offer their child. Therefore, it is likely that many parents are offering their infant more food than they need, particularly if they are using multi-serving packages, and if they rely on package size rather than infant satiety cues to decide when to stop offering food. In order to prevent overfeeding, it is important that parents and caregivers know how to recognise, and then respond appropriately to, the infant’s satiety cues, regardless of manufacturer-proposed serving sizes.

A major strength of the current study was that recipes were developed, which allowed the iron, vitamin B12, free sugars, and added sugars content to be estimated even though these are not usually reported on nutrition information panels. Other studies examining the nutritional quality of commercial infant foods have only had access to nutrient amounts provided on nutrition information panels or ingredient lists. In addition, comprehensive searches of commercial infant foods available in New Zealand were undertaken in four supermarkets that represent New Zealand’s two major supermarket chains. This gives greater confidence that the majority of the commercial infant food supply was captured.

A major limitation of the current study is that the specific proportions of ingredients in food products were not reported on all food labels and so had to be estimated (although recipes were carefully modeled on both the ingredient list and nutrition information panel). In addition, some ingredients were not available in FoodWorks so they had to be substituted with nutritionally similar foods or, in a very small number of instances, excluded. Analysing the foods in a laboratory would have been a more objective measure of nutrient content but was not feasible in this study.

## 5. Conclusions

In conclusion, this study shows that commercial infant food pouches contain similar amounts of energy, iron, vitamin B12, free sugars, and added sugars to other forms of commercial infant foods. Considerably more total sugars were found in pouches because pouches were more likely to include sweet fruits and vegetables. Parents and caregivers should be advised to minimise intake of sweet snacks and dairy foods because of their sugars content, and to ensure that if commercial infant foods are being used regularly, products from a range of food groups are offered, and not just fruit products. As infants age, and solid foods become a larger part of their diet, it is important that they move on quickly from fruit and vegetable purées to less processed (commercial or homemade) foods and replace their infant milk with a wider range of foods rather than simply increasing the portion size of sweet fruits and vegetables. The low iron content of pouches is of concern. Infants who are offered commercial infant food pouches must also be offered iron-rich foods from other sources to ensure their daily iron requirements are met [2].

Given the widespread concern about the sugars content of commercial infant foods, a universal definition of sugars that is appropriate for the infant diet is urgently needed so that sugars content and intakes can be monitored more effectively. This will help eliminate the confusion caused by the use of inconsistent definitions, which ultimately cause frustration for parents and caregivers. It is extremely important to agree on the status of fruit (and vegetable) juices, juice concentrates, pastes, powders, and purées in relation to the infant diet, and to ensure that the impact of commercial and home food preparation methods on the sweetness and availability of sugars in foods is investigated. Finally, research into the ways in which infant food pouches are being used in the community and the extent to which this has an impact on infant diet, growth, and dental health is crucially important. It is certainly possible that pouches are consumed in a different way and in different amounts to other commercial infant foods, but this can only be determined by direct observation of infants and families using them.

## Figures and Tables

**Table 1:** Food group classifications.

Food Group	Detail
Snack food (sub-groups: sweet snacks, savoury snacks)	Foods described as biscotti, biscuits, bites, cereal bars, dried fruits, fiddlesticks, freeze-dried fruits and vegetables, fruit snack bars, muesli fingers, nibblers, pops, puffs, rice biscuits, rice cakes, round-a-bouts, rusks, softcorn, wheels, yoghurt buttons and yoghurt drops.
Meat and fish	Contained meat, fish, or poultry.
Legume	Legume-based foods (e.g., hummus).
Breakfast cereal	Cereal-based foods that are ordinarily consumed as breakfast (e.g., cereal, muesli, oats, porridge), and infant cereals.
Dairy	Dairy-based foods (e.g., custard, yoghurt).
Vegetable	Vegetable-based foods (e.g., vegetable purées).
Fruit	Fruit-based foods (e.g., fruit purées, fruit salad).
Cereal, grains, and pasta	Foods based on cereals, grains, or pasta (e.g., spaghetti).

**Table 2:** Target age group classifications.

Age Group	Minimum Recommended Age Stated on the Packet
4–6 months	All ages (4–6 months onwards) From 4 months 4+ months 4–6+ months 5+ months
6–8 months	From 6 months 6+ months 6–7 months 7+ months
8–12 months	From 8 months 8+ months 9+ months 10+ months
12+ months	12+ months 1+ years 1–5 years 18+ months

**Table 3:** Description of pouch vs. non-pouch foods identified (*n* = 208) ^1^.

Characteristic	Pouch	Non-Pouch	Total ^2^
	*n* (%)	*n* (%)	*n* (%)
Total	133 (63.9%)	75 (36.1%)	208 (100.0%)
Age group			
4–6 months	35 (81.4%)	8 (18.6%)	43 (20.7%)
6–8 months	72 (81.2%)	16 (18.8%)	88 (42.3%)
8–12 months	19 (66.7%)	38 (33.3%)	57 (27.4%)
12+ months	7 (35.0%)	13 (65.0%)	20 (9.6%)
Food group			
Meat and fish	18 (35.3%)	33 (64.7%)	51 (24.5%)
Breakfast cereal	5 (27.8%)	13 (72.2%)	18 (8.7%)
Legume	9 (75.0%)	3 (25.0%)	12 (5.8%)
Dairy	24 (80.0%)	6 (20.0%)	30 (14.4%)
Vegetable	28 (66.7%)	14 (33.3%)	42 (20.2%)
Fruit	48 (90.6%)	5 (9.4%)	53 (25.5%)
Cereal, grains and pasta	1 (50.0%)	1 (50.0%)	2 (0.9%)

^1^ Snack foods (*n* = 58) are excluded. ^2^ Percentage totals are column totals adding to 100% each for “age group” and “food group”.

## Data Availability

The data presented in this study are available on reasonable request from the corresponding author.

## Supplementary Materials

The following are available online at https://www.mdpi.com/2072-6643/13/2/657/s1. Figure S1. Classification system to classify commercial infant foods into food groups. Figure S2. Energy content of foods in pouches vs non-pouches (values in kcal). Figure S3. Energy content of foods by form of food (values in kcal). Figure S4. Energy content of foods by food group (values in kcal). Figure S5. Energy content of foods by age group (values in kcal). Figure S6. Energy content of snack foods (values in kcal). Table S1. Percent energy from free sugars.
