# Supplementary material for: Energy, Sugars, Iron, and Vitamin B12 Content of Commercial Infant Food Pouches and Other Commercial Infant Foods on the New Zealand Market
Source: Nutrients. 2021 Feb 18;13(2):657. doi: 10.3390/nu13020657 (PMC7922386; doi:10.3390/nu13020657)
Supplement: Supplementary file 1 [file nutrients-13-00657-s001.zip › Figure S1.docx]

**Figure S1.** Classification system to classify commercial infant foods into food groups.

†For products that appeared to be legume-based and water was listed as the first or second ingredient on the ingredient list, it was assumed that the quantity of legume reported was the raw (i.e., uncooked) quantity. USDA conversion factors were applied to determine the cooked quantity of legume in the food [54]. The “kidney beans” conversion factor was used for products containing black beans or white navy beans because they did not have specific conversion factors.

**^*^**For products that appeared to be dairy-based and the quantity of milk was reported as a percentage of milk powder, the following ratios were used to reconstitute the milk powder to determine the equivalent quantity of milk in the final product: whole milk (1:8) and skim milk (1:10).

[54] Bowman S. A.; Martin C.L.; Carlson J.L.; Clemens J.C.; Lin B-H.; A.J., M. Food Intakes Converted to Retail Commodities Databases: 2003-08: Methodology and User Guide. U.S. Department of Agriculture, Agricultural Research Service, Beltsville, MD: 2013.
