# Supplementary material for: Energy, Sugars, Iron, and Vitamin B12 Content of Commercial Infant Food Pouches and Other Commercial Infant Foods on the New Zealand Market
Source: Nutrients. 2021 Feb 18;13(2):657. doi: 10.3390/nu13020657 (PMC7922386; doi:10.3390/nu13020657)
Supplement: Supplementary file 1 [file nutrients-13-00657-s001.zip › Figure S2-S6.docx]

**Figure S2.** Energy content of foods in pouches vs non-pouches (values in kcal). ^1 2^


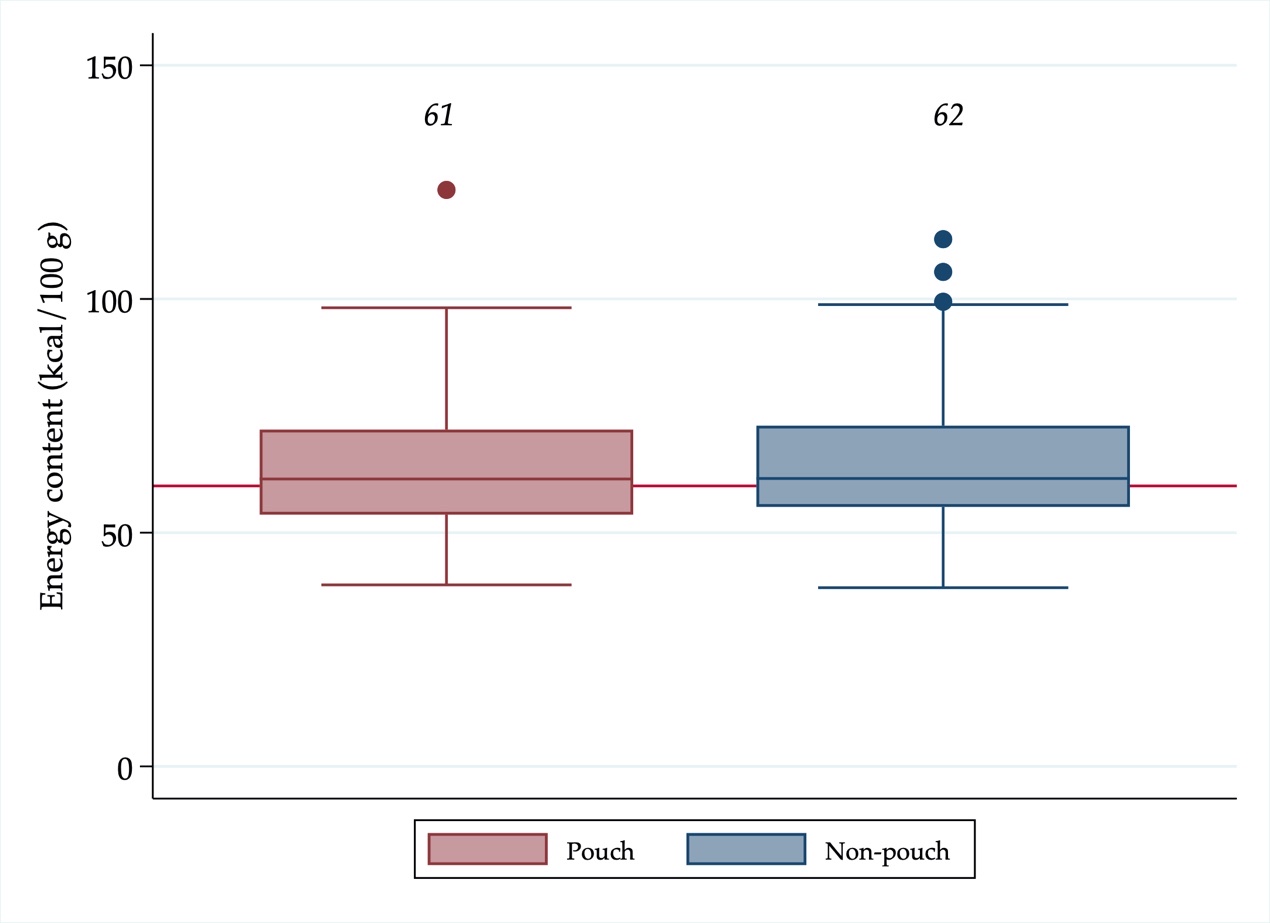


^1^ Median values are displayed above the boxes.

^2^ The line at the 60 kcal/100 g point in Figure S2 indicates the minimum energy density proposed by the WHO in their criteria for commercially available complementary foods that are considered suitable for infants and young children [27].

**Figure S3.** Energy content of foods by form of food (values in kcal). ^1 2^

**
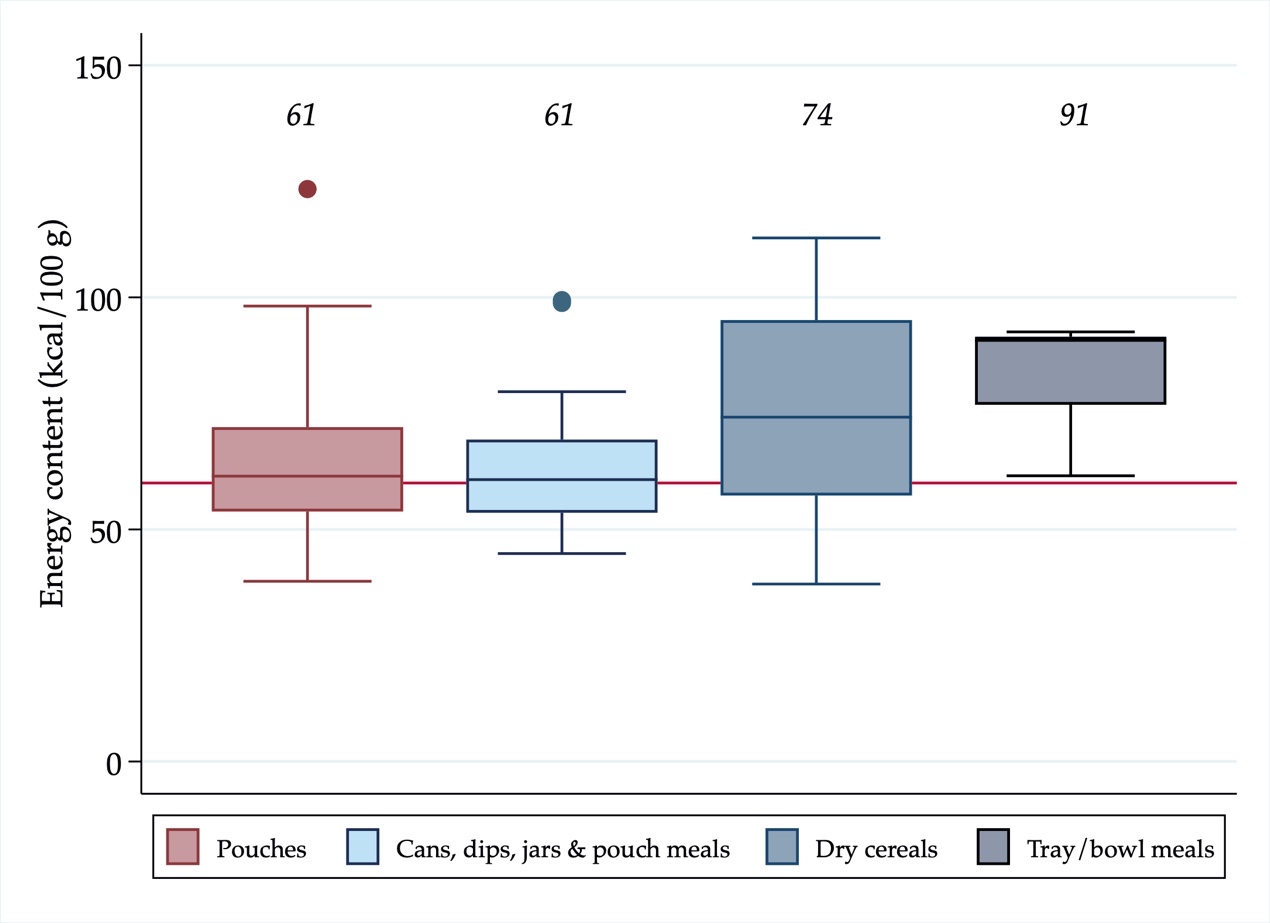
**

^1^ Median values are displayed above the boxes.

^2^ The line at the 60 kcal/100 g point in Figure S3 indicates the minimum energy density proposed by the WHO in their criteria for commercially available complementary foods that are considered suitable for infants and young children [27].

**Figure S4.** Energy content of foods by food group (values in kcal). ^1 2 3^

**
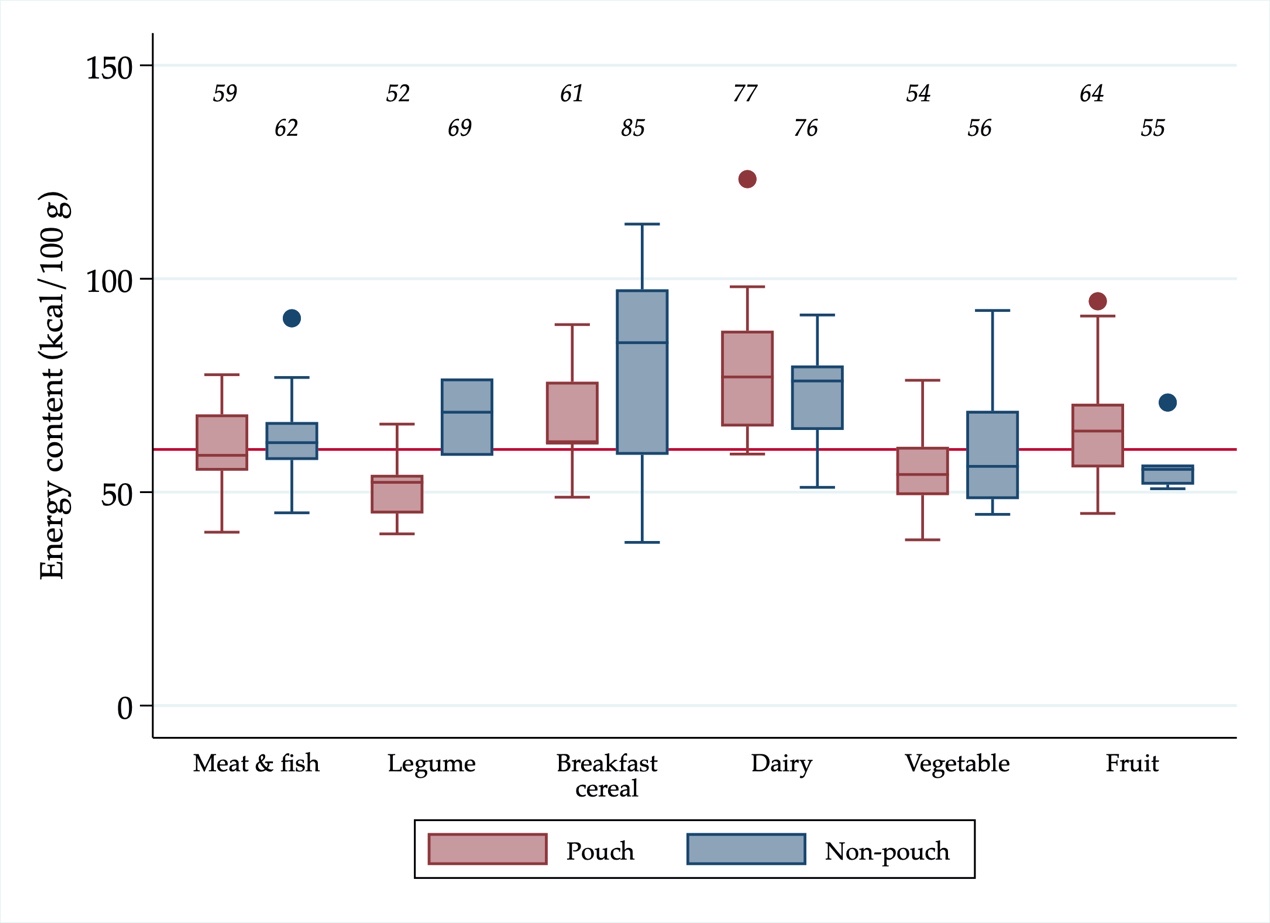
**

^1^ Median values are displayed above the boxes.

^2^ The line at the 60 kcal/100 g point in Figure S4 indicates the minimum energy density proposed by the WHO in their criteria for commercially available complementary foods that are considered suitable for infants and young children [27].

^3^ “Cereal, grains and pasta” food group not shown as only two foods were contained in the food group. This is because all other foods were classified into other food groups at higher levels of the classification system (see Figure S1).

**Figure S5.** Energy content of foods by age group (values in kcal). ^1 2^


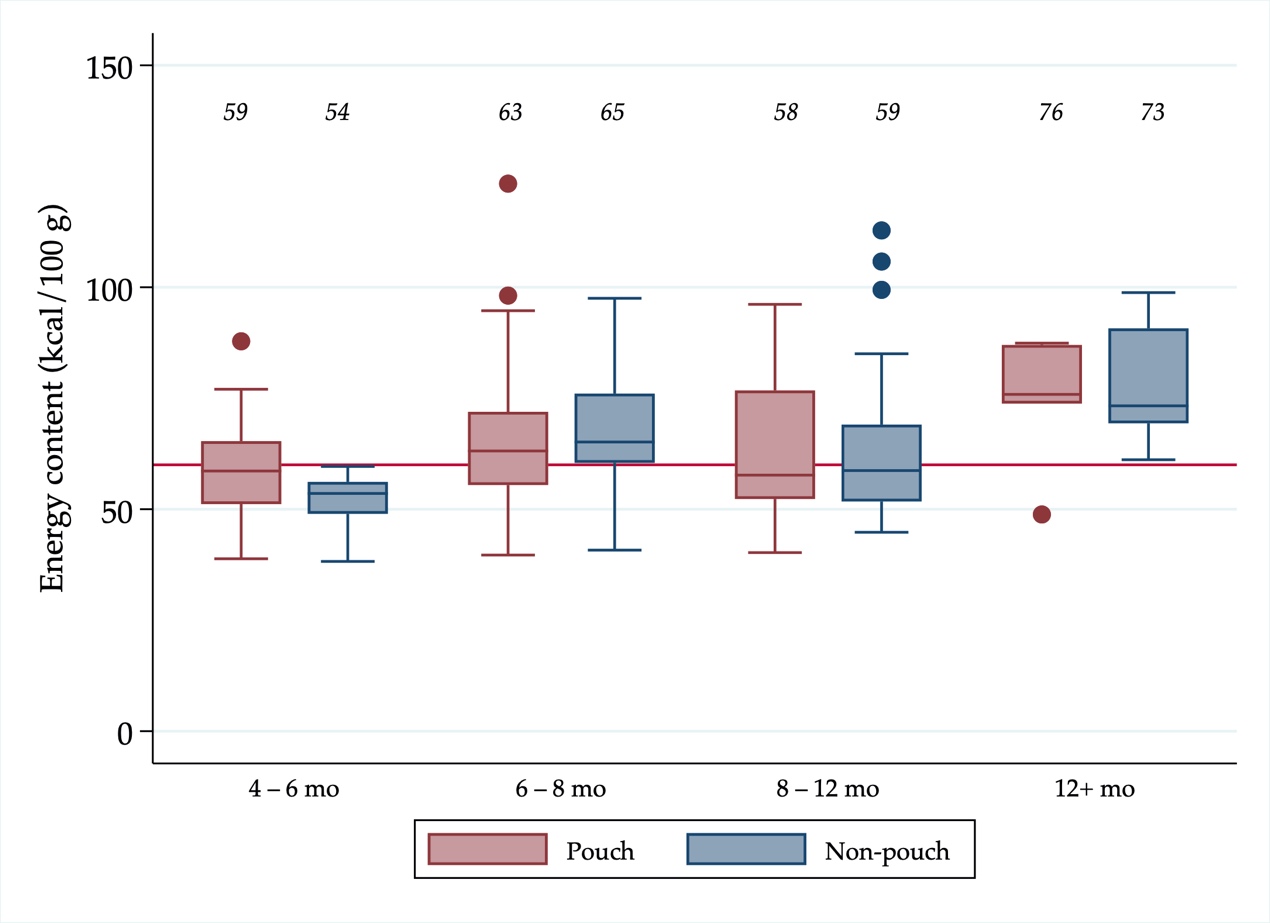


^1^ Median values are displayed above the boxes.

^2^ The line at the 60 kcal/100 g point in Figure S5 indicates the minimum energy density proposed by the WHO in their criteria for commercially available complementary foods that are considered suitable for infants and young children [27].

**Figure S6.** Energy content of snack foods (values in kcal). ^1^


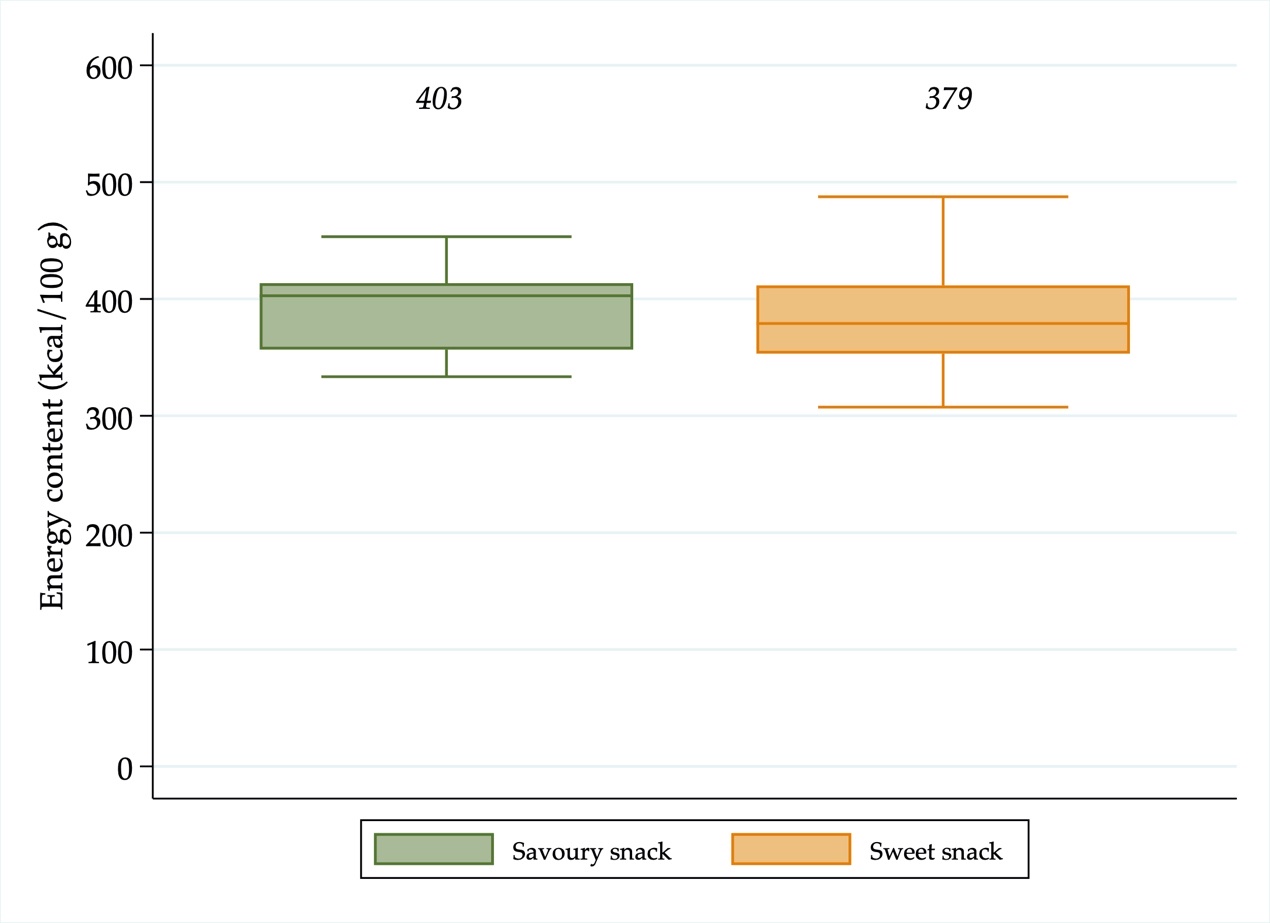


^1^ Median values are displayed above the boxes.
