# Supplementary material for: Energy, Sugars, Iron, and Vitamin B12 Content of Commercial Infant Food Pouches and Other Commercial Infant Foods on the New Zealand Market
Source: Nutrients. 2021 Feb 18;13(2):657. doi: 10.3390/nu13020657 (PMC7922386; doi:10.3390/nu13020657)
Supplement: Supplementary file 1 [file nutrients-13-00657-s001.zip › Table S1.docx]

**Table S1.** Percent energy from free sugars.

| **Characteristic** | **Pouch** | | **Non-pouch** | | **Total** |
| --- | --- | --- | --- | --- | --- |
|  | **Median (25^th^, 75^th^ percentile)** | | | | |
| Total | 0 (0, 0) | 0 (0, 8.7) | | 0 (0, 5.3) | |
| Age group ^1^ |  |  | |  | |
| 4–6 months | 0 (0, 0) | 0 (0, 0) | | 0 (0, 0) | |
| 6–8 months | 0 (0, 4.8) | 0 (0, 8.4) | | 0 (0, 4.8) | |
| 8–12 months | 0 (0, 24.1) | 0 (0, 0.9) | | 0 (0, 1.3) | |
| 12+ months | 0 (0, 0.4) | 1.3 (0, 1.6) | | 0.3 (0, 1.5) | |
| Food group ^2^ |  |  | |  | |
| Meat and fish | 0 (0, 1.0) | 0 (0, 1.5) | | 0 (0, 1.3) | |
| Breakfast cereal | 7.4 (0, 11.9) | 0 (0, 0.1) | | 0 (0, 1.0) | |
| Legume | 0 (0, 0) | 0 (0, 0) | | 0 (0, 0) | |
| Dairy | 25.6 (0.1, 29.1) | 17.8 (0.7, 30.4) | | 25.6 (0.3, 29.2) | |
| Vegetable | 0 (0, 0) | 0 (0, 0) | | 0 (0, 0) | |
| Fruit | 0 (0, 0.7) | 0 (0, 3.4) | | 0 (0, 1.3) | |
| Savoury snack | - | 0 (0, 3.1) | | 0 (0, 3.1) | |
| Sweet snack | - | 16.0 (0, 21.0) | | 16.0 (0, 21.0) | |

^1^ Snack foods are excluded from age group summary.

^2^ “Cereal, grains and pasta” food group not shown as only two products were contained in the food group. This is because all other foods were classified into other food groups at higher levels of the classification system (see Figure S1).
